# Follicular fluid-derived exosomal miR-143-3p/miR-155-5p regulate follicular dysplasia by modulating glycolysis in granulosa cells in polycystic ovary syndrome

**DOI:** 10.1186/s12964-022-00876-6

**Published:** 2022-05-09

**Authors:** Jianping Cao, Peng Huo, Kuiqing Cui, Huimei Wei, Junna Cao, Jinyuan Wang, Qingyou Liu, Xiaocan Lei, Shun Zhang

**Affiliations:** 1 Guangxi Key Laboratory of Environmental Exposomics and Entire Lifecycle Health, Guilin Medical University, Guilin, 541199 China; 2 State Key Laboratory for Conversation and Utilization of Subtropical Agro-Bioresources, Guangxi University, Nanning, 530004 Guangxi China; 3 Department of Reproductive Medical Center, The Affiliated Hospital of Guilin Medical University, Guilin, 541001 China; 4 Department of Histology and Embryology, Clinical Anatomy and Reproductive Medicine Application Institute, University of South China, Hengyang, 421001 China

**Keywords:** Polycystic ovary syndrome, Exosomes, Glycolysis, miR-143-3p, miR-155-5p, HK2

## Abstract

**Objective:**

Polycystic ovary syndrome (PCOS) is characterized by follicular dysplasia. An insufficient glycolysis-derived energy supply of granulosa cells (GCs) is an important cause of follicular dysplasia in PCOS. Follicular fluid (FF) exosomal microRNAs (miRNAs) have been proven to regulate the function of GCs. In this study, exosomes extracted from clinical FF samples were used for transcriptome sequencing (RNA-seq) analysis, and a human ovarian granulocyte tumour cell line (KGN cells) was used for in vitro mechanistic studies.

**Methods and results:**

In FF exosomal RNA-seq analysis, a decrease in glycolysis-related pathways was identified as an important feature of the PCOS group, and the differentially expressed miR-143-3p and miR-155-5p may be regulatory factors of glycolysis. By determining the effects of miR-143-3p and miR-155-5p on hexokinase (HK) 2, pyruvate kinase muscle isozyme M2 (PKM2), lactate dehydrogenase A (LDHA), pyruvate, lactate and apoptosis in KGN cells, we found that upregulated miR-143-3p expression in exosomes from the PCOS group inhibited glycolysis in KGN cells; knockdown of miR-143-3p significantly alleviated the decrease in glycolysis in KGN cells in PCOS. MiR-155-5p silencing attenuated glycolytic activation in KGN cells; overexpression of miR-155-5p significantly promoted glycolysis in KGN cells in PCOS. In this study, HK2 was found to be the mediator of miR-143-3p and miR-155-5p in FF-derived exosome-mediated regulation of glycolysis in KGN cells. Reduced glycolysis accelerated apoptosis of KGN cells, which mediated follicular dysplasia through ATP, lactate and apoptotic pathways.

**Conclusions:**

In conclusion, these results indicate that miR-143-3p and miR-155-5p in FF-derived exosomes antagonistically regulate glycolytic-mediated follicular dysplasia of GCs in PCOS.

**Video Abstract**

**Supplementary Information:**

The online version contains supplementary material available at 10.1186/s12964-022-00876-6.

## Introduction

Polycystic ovary syndrome (PCOS) is a common endocrine disease characterized by dysplasia of follicles, sparse ovulation or anovulation, and hyperandrogenaemia in women of childbearing age worldwide and affects up to 10% of women [1]. The prevalence of infertility is up to 70% in patients with PCOS, which is often accompanied by type II diabetes, obesity, and endometrial cancer [2–4]. Women with PCOS suffer from widespread depression and anxiety, and PCOS can exert long-term harmful effects on women's physical and mental health [5]. Although the clinical manifestations and biochemical indicators of patients with PCOS are highly heterogeneous, follicular dysplasia is still a typical feature of PCOS, affecting approximately 10% of infertile women [6].

Follicular fluid (FF) provides the microenvironment for follicular development, and changes in the follicular microenvironment are the main cause of follicular dysplasia [7]. FF is filled with a variety of vesicles and molecules, including exosomes, which largely contribute to follicular development and ovum maturation [8]. Studies have proven that some nucleic acids and proteins in FF are closely involved in follicular glucose metabolism, lipoprotein metabolism, cell proliferation and other processes in patients with PCOS [9–11]. FF-derived exosomes are the main carriers of these nucleic acids and proteins. However, the role of FF-derived exosomes in follicular dysplasia in PCOS remains unknown.

The discovery of exosomes as carriers of noncoding RNAs, such as microRNAs (miRNAs) and mRNAs, largely facilitates the rapid development of exosomes as the transport medium of genetic information in the study of various diseases [12, 13]. Exosomes are highly stable and exist stably in various body fluids or cell culture medium supernatants. Studies have proven that the RNA composition of exosomes is different from that of cells secreting exosomes, but the expression of miRNAs of exosomes and secreting exosomes is highly similar [14–16]. In cancer cells, exosomal miRNAs play an important role in signal transmission between cells and can modulate cell migration, cell differentiation, immune response, antigen presentation, tumour invasion and so on [17]. To the best of our knowledge, only a few studies have been performed to examine the functional role of FF-derived exosomal miRNAs. FF-derived exosomes are mainly secreted by granulosa cells (GCs). Recent studies have demonstrated that FF-derived exosomal miRNAs regulate the function of GCs in patients with PCOS [18]. Energy is the primary condition for follicular development. Several studies have proven that patients with PCOS often have abnormal glucose metabolism, and glycolysis of GCs is the main energy source for follicular development [19]. Lactate, the glycolytic product of GCs, is an environmental stimulus for follicular development, and apoptosis of GCs has been proven to be the main reason for follicular atresia in PCOS [19]. Thus, glycolysis of GCs is crucial for the follicular development of PCOS. At present, studies on the regulatory factors of glycolysis in GCs mainly focus on endocrine hormones, signalling pathways and nonfollicular fluid miRNAs [20–22]. To the best of our knowledge, the regulation of glycolysis in GCs by FF-derived exosomal miRNAs has not yet been studied. Therefore, exploring the role of FF-derived exosomal miRNAs may reveal novel mechanisms for follicular dysplasia of PCOS.

In this study, exosomes extracted from clinical FF samples were used for transcriptome sequencing analysis, and a human ovarian granulocyte tumour cell line (KGN cells) was used for the in vitro mechanistic studies. In this study, we reveal the proposed potential therapeutic targets and mechanisms by which exosomal miR-143-3p and miR-155-5p antagonize glycolysis in PCOS-related follicular dysplasia. The present study may provide novel insights into the pathophysiology of PCOS.

## Materials and methods

### Patients

This study was approved by the Ethics Committee of the Affiliated Hospital of Guilin Medical College, and all samples were obtained with informed consent from patients. The present study included 6 women with PCOS and 6 women without PCOS who underwent in vitro fertilization-embryo transfer (IVF-ET) in the Reproductive Center of The Affiliated Hospital of Guilin Medical College from January 2020 to June 2020. For the PCOS group, the Rotterdam Consensus (2004) was used to define the phenotype of the patients with PCOS who met at least two of the following criteria: (1) sparse ovulation or anovulation (menstruation more than 35 days or menstruation less than 8 times per year); (2) signs of hyperandrogenism (evidence of increased circulating total testosterone of more than 45 ng/dL or hypertrichosis); and (3) polycystic characteristics with more than 12 follicles, all of which were less than 9 mm in diameter, as shown by ultrasonic monitoring of ovarian morphology. Other causes, such as delayed adrenocortical hyperplasia and adrenal androgen-secreting neoplasms, were excluded. The inclusion criteria of the control group were normal ovarian morphology and function, regular menstrual cycle (26–35 days), normal androgen level (< 45 ng/dL), and 6–10 antrum eggs on both sides. The exclusion criteria for both groups were as follows: age over 35 years, ovarian cysts and tumours, history of ovarian surgery or radiotherapy and chemotherapy, endometriosis, hyperprolactin, thyroid dysfunction and chromosomal abnormalities. Patients who took drugs affecting hormone levels or glucose and lipid metabolism within six months prior to treatment were also excluded.

### Isolation and characterization of FF-derived exosomes

Baseline hormone levels were measured before induction of ovulation all patients, and ovulation was stimulated by a short-effect and long-duration regimen in the luteal phase. After 36 h of triggering by human chorionic gonadotropin (hCG) injection (2000 U, Lizong Medicine Factory, Zhuhai, China), serum-free FF with bilateral follicles with a diameter of > 15 mm was collected by aspiration. After centrifugation at 1500 g for 15 min, the supernatant was taken for preservation. The exosomes of FF were extracted by ultracentrifugation in strict accordance with the instructions of the Qiagen exoEasy Maxi Kit (Qiagen, Hilden, Germany). The positive marker proteins CD63 (#A19023; 1:1000 dilution, Abcam, Cambridge, UK) and CD9 (#19027; 1:1000 dilution, Abcam) were detected by Western blot assays.

The morphology of exosomes was identified by transmission electron microscopy (TEM). The phosphate-buffered saline (PBS)-dissolved exosomes were prefiltered with a 0.2 µm aqueous phase filtration membrane. The precipitation sample of 20 µl of PBS was resuspended and dropped onto the copper wire and stained with 2% phosphotungstic acid (pH 5.0) for 60 s. The morphology of exosomes was observed by transmission electron microscopy (JEOL, Tokyo, Japan). The particle size and concentration of exosomes were analysed by nanoparticle tracking (NTA), and the exosome suspension was diluted to 800 mL with PBS (1:200) and injected into the sterile sample pool. The Browne motion trajectory of captured exosomes was observed by a Nanosight NS300 on the computer, and the diameter of exosome particles was measured by Zetaview in NTA2.1.

### Exosomal RNA analyses

RNA sequencing (RNA-seq) of FF-derived exosomes was performed by Aksomics (Shanghai, China). The total RNA samples were examined and quantified by agarose electrophoresis and Nanodrop, and the library quality was determined by an Agilent 2100 Bioanalyzer. Sequencing libraries of different samples were mixed and denatured into single-stranded DNA with 0.1 M NaOH. The original amplified clusters were captured on the Illumina Flow cell and cycled on an Illumina NextSeq 500 sequencer according to the instructions. The data generated by Illumina NextSeq 500 are raw sequencing data, which were evaluated by quality control to determine whether the sequencing data can be used for subsequent data analysis. Crosstalk data from crosstalk and crosstalk removed from reads were compared with Bowtie to the reference genome GRCh38 after quality control and were analysed statistically if the crosstalk results were favourable. RNAs with counts per million mean ≥ 1 were considered to be expressed in groups and statistically analysed. EdgeR was used for analysis of intergroup differences. Differentially expressed RNAs were screened by log2|fold change (FC)|≥ 0.585 and P value < 0.05 for cluster analysis. The reagents used above included the NEBNext ® Poly(A) mRNA Magnetic Isolation Module (New England Biolabs, Ipswich, USA); the RiboZero Magnetic Gold Kit (Human/Mouse/Rat) (Epicentre, an Illumina Company, San Diego, USA); the NEB Multiplex Small RNA Library Prep Set for Illumina; and the TruSeq Rapid SR Cluster Kit (#GD-402-4001, Illumina, San Diego, USA). For the convenience of description, we named 6 exosome samples in the control group C1-C6 and 6 exosome samples in the PCOS group T1-T6. To explore whether the differentially expressed genes in the FF-derived exosomes between the control and PCOS groups are involved in glycolysis of GCs, we screened the Gene Ontology (GO) terms and Kyoto Encyclopedia of Genes and Genomes (KEGG) pathways for the combined enrichment of differentially expressed miRNA and mRNA with glucose metabolism as the core via differential enrichment analysis. GO term and KEGG pathway enrichment (http://www.genome.jp/kegg/) analysis of differentially expressed genes (DEGs) was achieved by clustering Profiler R.

### Cell culture and transfection

KGN cells were a generous gift from the Institute of Applied Anatomy and Reproductive Medicine, Hengyang Medical College, University of South China. The KGN cells were cultured with high glucose Dulbecco's modified Eagle’s medium (DMEM, #11965084, Gibco, Waltham, USA) with 10% foetal bovine serum (FBS, #10099141C, Gibco) in a humidified atmosphere with 5% CO_2_ at 37 °C. For the in vitro cellular model of PCOS, KGN cells were cultured with high glucose DMEM with 10% foetal bovine serum (FBS) and 100 nM testosterone (#M6105, AbMole BioScience, Houston, USA) in a humidified atmosphere with 5% CO_2_ at 37 °C.

The miR-143-3p mimics, miR-155-5p mimics, miR-143-3p inhibitor and miR-155-5p inhibitor were purchased from RiboBio (Guangzhou, China). Cell transfection was performed using the riboFECT CP Transfection Kit (#C10511-05, RiboBio) when the cells reached 50–60% confluence. The transfection efficiency was evaluated by a riboMONITOR Transfection Indicator Kit (#C10410-5, RiboBio) at 12 h after transfection.

### Luciferase reporter assay

A wild-type (WT) or mutant (MUT) mRNA fragment was constructed and inserted downstream of the luciferase reporter gene of psiCheck2 (#C8021, Promega, Madison, USA). When KGN cells reached 50–60% confluence, miRNA mimics (100 nM) or the mimic control and psiCheck2-mRNA-3′ untranslated region (3′ UTR)-MUT (15 mg/L) or psiCheck2-mRNA-3′ UTR-MUT (15 mg/L) were cotransfected into KGN cells by ExFect Transfection Reagent (#T101-01, Vazyme, Nanjing, China). At 36 h after transfection, the activities of Renilla luciferase and firefly luciferase were determined by the Dual-luciferase Reporter Assay System (#E1910, Promega). Renilla luciferase activity was normalized to firefly luciferase activity, and relative luciferase activity was calculated.

### Quantitative real-time PCR (qPCR)

RNA extraction was performed by using the TRIzol (#15596026, TRIzol™, Thermo Fisher, Waltham, USA) method according to the manufacturer’s protocol, and the stem-loop method was used to reverse transcribe miRNA into cDNA. Total RNA was reverse transcribed into cDNA according to HiScript III-RT SuperMix for qPCR (#R323-01, Vazyme) instructions. qPCR analysis was performed on an Applied Biosystems QuantStudio (#A34321, Thermo Fisher Scientific) by using ChamQ Universal SYBR qPCR Master Mix (#Q711-02, Vazyme). β-actin was used as an internal reference, and the gene expression level was calculated by the comparative 2^−△△Ct^ method. The primer sequences are shown in Table 1.

**Table 1:** Primers for real-time quantitative PCR

Gene	5′–3′	Amplicon size
β-Actin	F: GTGGCCGAGGACTTTGATTG	73
	R: CCTGTAACAACGCATCTCATATT	
HK2	F:CGACAGCATCATTGTTAAGGAG	233
	R: GCAGGAAAGACACATCACATTT	
PKM2	F: ACTGGCATCATCTGTACCATTG	84
	R: AGCCACATTCATTCCAGACTTA	
LDHA	F: CCAATATGGCAACTCTAAAGGATC	151
	R: GCAAGTTCATCTGCCAAGTCCT	
Bcl2	F: GACTTCGCCGAGATGTCCAG	129
	R: GAACTCAAAGAAGGCCACAATC	
Bax	F: CGAACTGGACAGTAACATGGAG	157
	R: CAGTTTGCTGGCAAAGTAGAAA	
hsa-miR-143-3p	RT: GTCGTATCCAGTGCAGGGTCCGAGGTATTCGCACTGGATACGACGAGCTA	65
	F: TGAGATGAAGCACTGTAGCTCGTC	
	R: CAGTGCAGGGTCCGAGGTAT	
hsa-miR-155-5p	RT: GTCGTATCCAGTGCAGGGTCCGAGGTATTCGCACTGGATACGACAACCCC	65
	F: GCGGCTCCTACATATTAGCATTAAC	
	R: CAGTGCAGGGTCCGAGGTAT	

*RT* specific primes corresponding to miRNA

### Western blot analysis

The protein samples were extracted using ice-cold radioimmunoprecipitation assay buffer (#89901, Thermo Fisher Scientific). The protein concentration was determined by a bicinchoninic acid assay kit (#PC0020, Solarbio, Beijing, China). Denatured proteins were separated by 10% sodium dodecyl sulphate–polyacrylamide gel electrophoresis (#1610173, TGX FastCast Acrylamide Kit, Bio-Rad, Hercules, USA) and then transferred to polyvinylidene membranes. The membrane was blocked with Tris-buffered saline with 0.1% Tween 20 (TBST) containing 5% skim milk powder for 2 h. After the membrane was washed with TBST, it was incubated with primary antibodies against hexokinase (HK) 2 (#A20829, 1:1000 dilution, ABclonal, Woburn, USA), pyruvate kinase muscle isozyme M2 (PKM2, #4053, 1:1000 dilution, Cell Signaling Technology, Danvers, USA), lactate dehydrogenase A (LDHA, #3582, 1:1000 dilution, Cell Signaling Technology) and β-actin (#3700, 1:1000 dilution, Cell Signaling Technology) overnight at 4 °C. Then, the membrane was incubated with horseradish peroxidase (HRP)-conjugated goat anti-mouse IgG (H + L) (#AS003, 1:5000 dilution, ABclonal) or HRP-conjugated goat anti-mouse IgG (H + L) (#AS014, 1:5000 dilution, ABclonal) at room temperature for 2 h. Finally, the protein chromogenic bands were detected by the Tanon 5500 chemiluminescence imaging system by using an enhanced chemiluminescence kit (#CW0049, CWBIO, Boston, USA) and analysed by ImageJ (https://imagej.nih.gov/ij/).

### Determination of pyruvate and lactate levels

At 36 h after different treatments, pyruvate and lactate concentrations in culture medium were determined by a pyruvate determination kit (#A081-1-1, Nanjing Jiancheng Bioengineering Institute, Nanjing, China) and lactate test kit (#A019-2-1, Nanjing Jiancheng Bioengineering Institute), respectively. The culture media of the different processed cells were collected in a 1.5 ml centrifuge tube and photographed (Table 2).

**Table 2:** Clinical characteristics of participants by study group

Parameters	PCOS group n = 6	Control group n = 6	Difference between groups^a^		
	Mean (± SD)	Mean (± SD)	Adjusted mean difference (95%CI)^b^	p value	q value
Age	30.250 (4.3725)	27.429 (2.507)	0.71 (− 0.26 to 1.67)	0.139	0.175
Years of infertility	3.750 (1.960)	2.429 (0.976)	0.75 (− 0.22 to 1.72)	0.116	0.168
Body mass index (BMI)^c^	23.390 (2.131)	22.366 (2.202)	0.45 (− 0.49 to 1.40)	0.332	0.319
Luteinizing hormone (LH) IU/L^d^	13.077 (3.741)	5.029 (1.924)	2.39 (1.12 to 3.65)	< 0.001	< 0.001
Follicle stimulating hormone (FSH) IU/L^d^	5.178 (1.303)	6.410 (1.903)	− 0.76 (− 1.73 to 0.21)	0.111	0.168
LH:FSH ratio	2.514 (0.399)	0.804 (0.278)	4.52 (2.66 to 6.39)		< 0.001
Estradiol (E2) pmol/L^d^	147.4 (38.756)	140.057 (146.376)	0.08 (− 0.86 to 1.01)	0.870	0.678
Testosterone nmol/L	2.023 (0.287)	0.697 (0.334)	4.16 (2.41 to 5.92)	< 0.001	< 0.001
Prolactin (PRL)mIU/L^d^	349.016 (214.871)	363.9 (132.897)	− 0.08 (− 1.02 to 0.87)	0.873	0.678
Anti-mullerian hormone (AMH)ng/ml	9.933 (5.628)	4.450 (1.800)	1.13 (0.11 to 2.14)	0.024	0.048
Progesterone (P)nmol/L	0.452 (0.275)	0.657 (0.653)	− 0.44 (− 1.38 to 0.51)	0.347	0.319
Fasting glucose nmol/L	5.023 (0.412)	5.216 (0.296)	− 0.49 (− 1.44 to 0.46)	0.294	0.319
Sinus follicle number	32.167 (10.098)	20.857 (4.981)	1.25 (0.22 to 2.28)	0.014	0.034

^a^All results are reported after adjustment for baseline values using ANCOVA

^b^Intervention effects are quantified as differences between groups in the mean value for each scale, estimated using linear regression models that adjusted for recruitment site and language of interview

^c^BMI = bodyweight (kg) divided by height (metres) squared. (BMI = w(kg)/height(m)^2^)

^d^Log transformations were carried out on the data before analyses

### EdU assay

At 36 h after different treatments, cells were stained with a Cell-Light EdU Apollo567 In Vitro Kit (#C10310-1, RiboBio) according to the manufacturer’s instructions. EdU-positive signals were detected by the EVOS (#AMF7000, Thermo Fisher Scientific) imaging system (40X) and were analysed by ImageJ (https://imagej.nih.gov/ij/).

### Statistical analysis

Data were analysed using GraphPad Prism 8.0 and IBM SPSS Statistics 26. The data are presented as the mean ± standard deviation (SD). Significant differences between/among different treatment groups were analysed by using an unpaired t test or one-way analysis of variance (ANOVA) followed by Bonferroni’s multiple comparison tests. *p < 0.05, **p < 0.01 and ***p < 0.001 were considered statistically significant.

## Results

### Isolation and identification of FF-derived exosomes

First, we compared the clinical information of patients between the control and PCOS groups. Baseline hormone levels were measured before induction of ovulation, and there were no significant differences in age, years of infertility, body mass index, follicle stimulating hormone, prolactin, progesterone or fasting blood glucose between the control and PCOS groups. The luteinizing hormone, anti-Mullerian hormone, testosterone and sinus follicle numbers in the patients with PCOS were significantly higher than those in the control group.

**Fig. 1:**
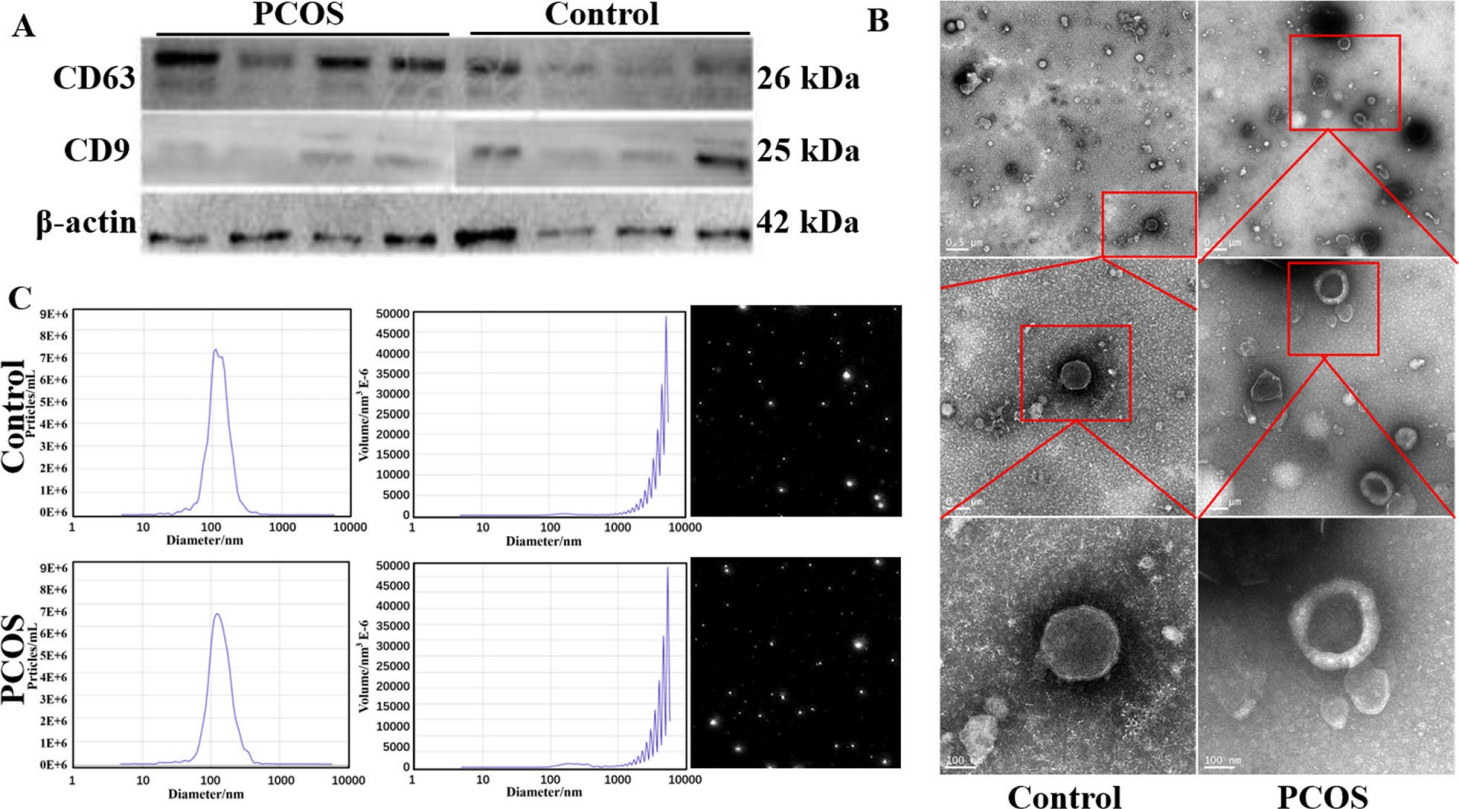
Characterization of FF-derived exosomes (**A**) representative western blotting of FF exosomal positive marker proteins (CD9, CD63) isolated from the control and PCOS groups. **B** Transmission electron microscopy (TEM) of FF exosomes isolated from the control group and PCOS group. **C** Representative nanoparticle tracking analysis (NTA) of exosomes isolated from the control and PCOS groups

Then, we further extracted exosomes from the FF of the patients from the control and PCOS groups. To ensure the quality of the exosomes, we characterized exosomes by using Western blotting, TEM and NTA. The Western blot assay results showed that exosome-positive marker proteins (CD63 and CD9) were expressed in all samples (Fig. 1A). TEM showed that exosomes were disc-shaped vesicles (a diameter of 100–150 nm, intact structure and high purity) with clear edges and light staining in the centre (Fig. 1B). The peak diameter of the exosomes measured by NTA was approximately 130 nm, and the concentration of exosomes was approximately 8 × 10^10^ particles/ml (Fig. 1C).

### RNA sequencing analysis of differentially expressed exosomal RNAs

**Fig. 2:**
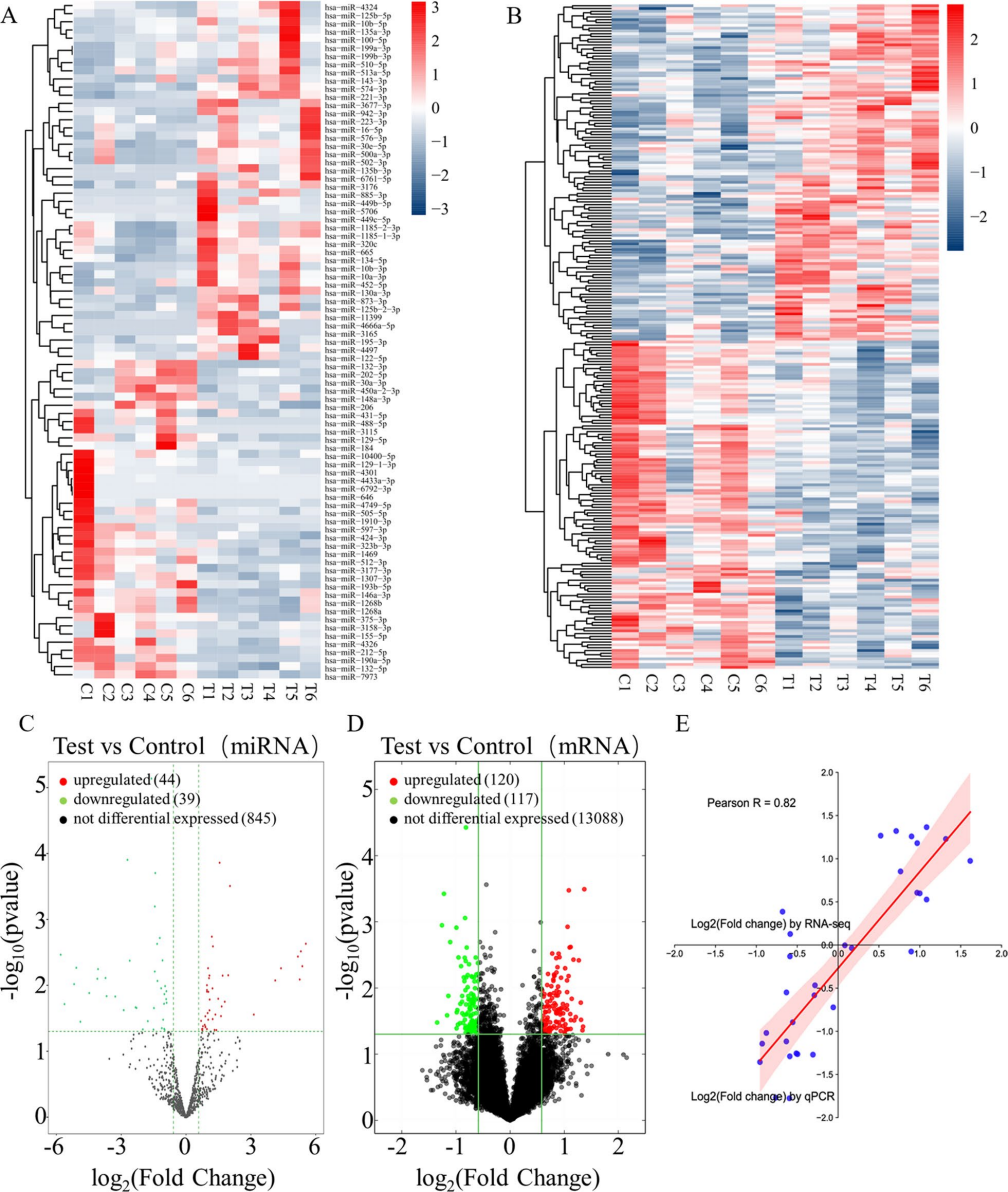
Exosomal RNA-seq data of the control group and PCOS group. C1–C6 were 6 random samples from the control group, and T1–T6 were 6 random samples from the PCOS group. **A** Heatmap showing that miRNAs were differentially expressed between the control and PCOS groups. **B** Heatmap showing hierarchical cluster analysis of mRNAs in the control and PCOS groups. **C** Volcano plot of differentially expressed miRNAs. **D** Volcano plot of differentially expressed mRNAs. **E** Correlation of expression changes observed by RNA-seq (Y-axis) and qPCR (X-axis)

From the deep RNA-seq sequencing results, we collected 128,005,498 reads, with > 94.54% base quality scores exceeding Q30 and a crosstalk ratio with the reference genome ≈ 60%. A total of 928 miRNAs and 13425 mRNAs were detected. A total of 83 differentially expressed miRNAs (44 upregulated and 39 downregulated) in the FF-derived exosomes were detected between the control and PCOS groups (Fig. 2A, C). A total of 337 differentially expressed mRNAs (120 upregulated and 117 downregulated) in the FF-derived exosomes were detected between the control and PCOS groups (Fig. 2B, D). To verify the reliability of the RNA-seq data, we randomly selected 32 RNAs for qPCR analysis and found that the correlation coefficient of the fold change in gene expression levels between the RNA-seq and RNA-qPCR results was high (R^2^ = 0.6724, P < 0.001), indicating good reliability of the RNA-seq data (Fig. 2E).

### FF-derived exosomal miRNAs and mRNAs regulate glycolysis of GCs in PCOS

**Fig. 3:**
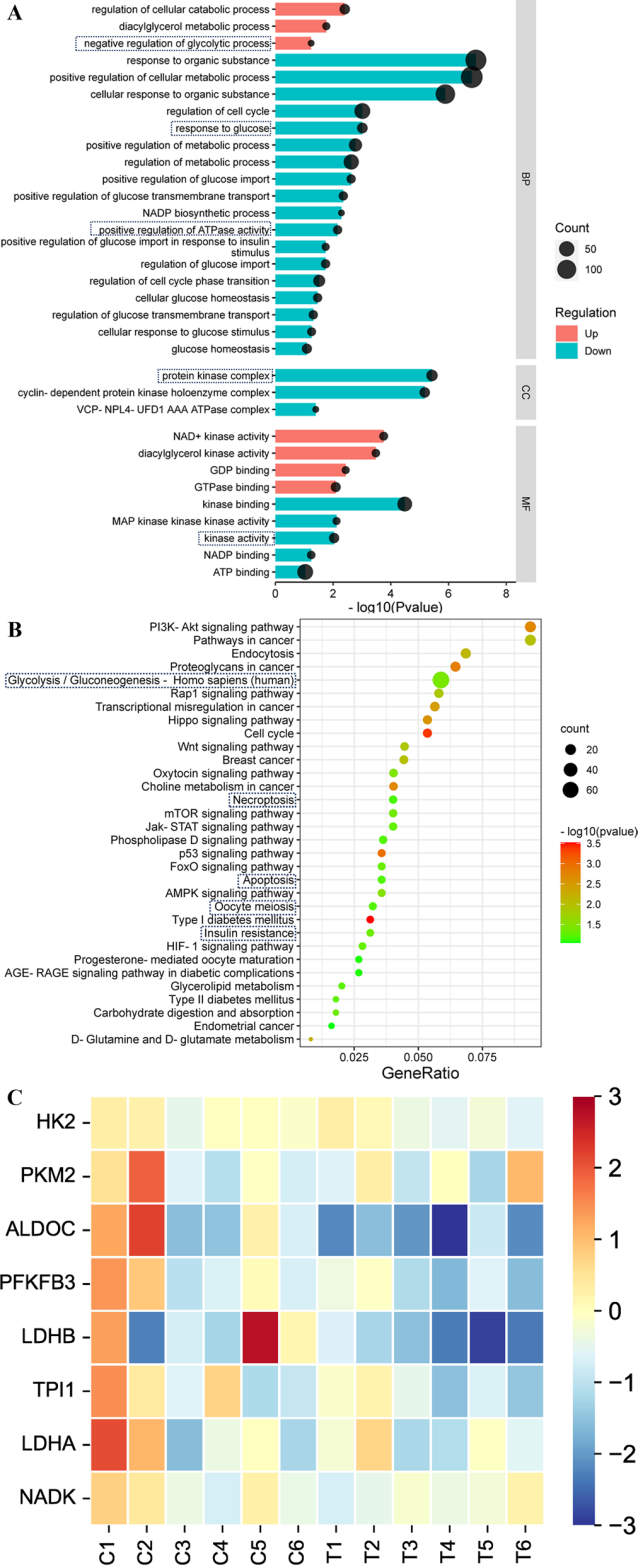
Enrichment analysis of differentially expressed genes in exosomal RNA-seq. **A** GO (Gene Ontology) analysis of differentially expressed miRNAs and mRNAs. The colour of the column indicates upregulation or downregulation, and the size of the dot indicates the number of genes, as shown on the right. **B** KEGG enrichment analysis of differentially expressed miRNAs and mRNAs. The colour and size of the dots represent − log10 (p value) and gene number, respectively, as shown on the right. **C** Heatmap of differentially expressed genes in the glycolytic pathway

Compared with those in the control group, the negative regulation of exosomal RNAs on glycolysis increased significantly in the PCOS group, and the processes related to glycolysis and energy metabolism, such as glucose import and response, protein kinase synthesis and activity, and positive regulation of ATPase activity, decreased significantly (Fig. 3A). In addition, there were significantly enriched pathways related to apoptosis, such as the hypoxia-inducible factor-1 signalling pathway, insulin resistance, oocyte meiosis and necrotic apoptosis, in the KEGG pathways jointly regulated by miRNAs and mRNAs. In addition, a cluster of differentially expressed genes with the largest number of miRNAs and mRNAs in the FF-derived exosomes between the control and PCOS groups was enriched in the glycolytic pathway (Fig. 3B). Furthermore, we analysed the expression of glycolytic genes in the RNA-seq results and found that the expression of key glycolytic genes in the PCOS group was significantly lower than that in the control group (Fig. 3C). These results suggest that there is a mechanism for significant changes in the glycolytic capacity and apoptotic state in exosomes from the PCOS group, which are coregulated by miRNAs and mRNAs. It has been reported that FF-derived exosomal miRNAs mainly regulate mRNA to affect the function of GCs [18]. Therefore, we speculate that GCs are the target of glycolysis regulated by FF-derived exosomal miRNAs. Thus, we explored whether FF-derived exosomal miRNAs regulated glycolysis in KGN cells in vitro.

### Differentially expressed exosomal miR-143-3p and miR-155-5p antagonize HK2 in KGN cells

To clarify the potential regulatory mechanism of exosomal miRNAs in glycolysis of GCs, we analysed the interactions of differentially expressed miRNAs in Fig. 2A and glycolytic genes in Fig. 3C through miRBD (http://mirdb.org/) and TargetScan (http://www.targetscan.org/vert_71/). We found that the upregulated miR-143-3p expression in exosomes from the PCOS group was a potential regulatory factor of HK2, while downregulated miR-155-5p expression was confirmed to negatively regulate the transcriptional activator C/EBPβ of miR-143-3p in a variety of cells and tissues [23, 24].

**Fig. 4:**
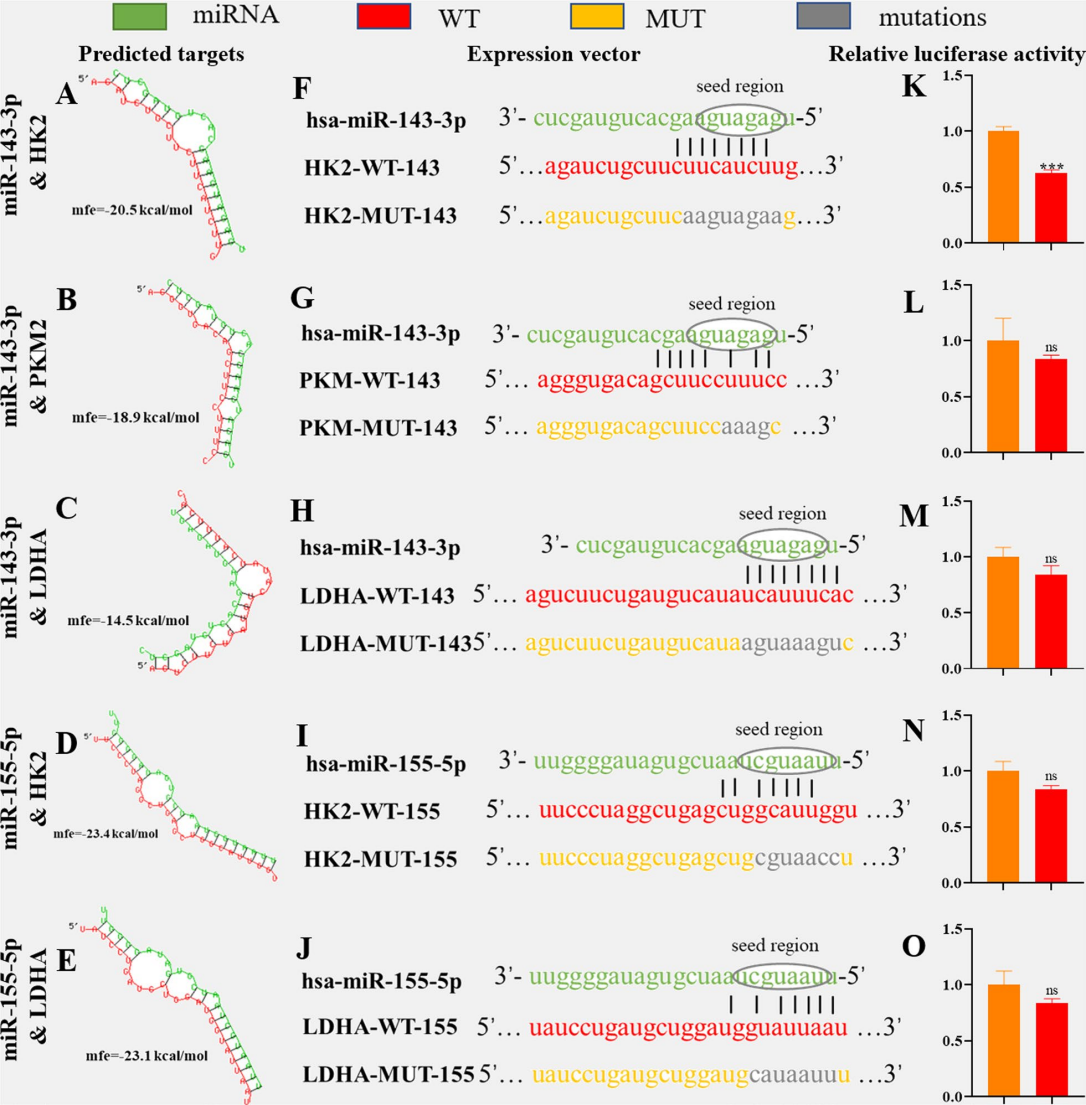
The interaction between miRNAs and the targeted genes. **A**–**E** Prediction targets between miR-143-3p/miR-155-5p and key glycolytic genes (HK2, PKM2, LDHA). **F**–**J** Luciferase reporter vectors. **K–O** Inhibitory rate of miRNA on luciferase activity of WT and MUT. mfe (hsa-miR-143-3p) = −43.8 kcal/mol, mfe (hsa-miR-155-5p) = −46.39 kcal/mol (*p < 0.05 and ***p < 0.001)

To identify the possible targets between miR-143-3p/miR-155-5p and glycolysis, we used RNAhybrid (https://bibiserv.cebitec.uni-bielefeld.de/rnahybrid/) to detect potential targets of key glycolytic genes (Fig. 4A–E). We constructed WT vectors and MUT vectors of the luciferase reporter for each potential target (Fig. 4F–J). Through the cotransfection of miRNA and vector into KGN cells, we found that only miR-143-3p showed a significant relationship with HK2 (Fig. 4K–O). Therefore, HK2 is the only regulatory mediator of miR-143-3p/miR-155-5p and glycolysis. MiR-143-3p silenced HK2, and miR-155-5p activated HK2 by silencing miR-143-3p.

### Expression of miR-143-3p/miR-155-5p in the cellular model of PCOS

**Fig. 5:**
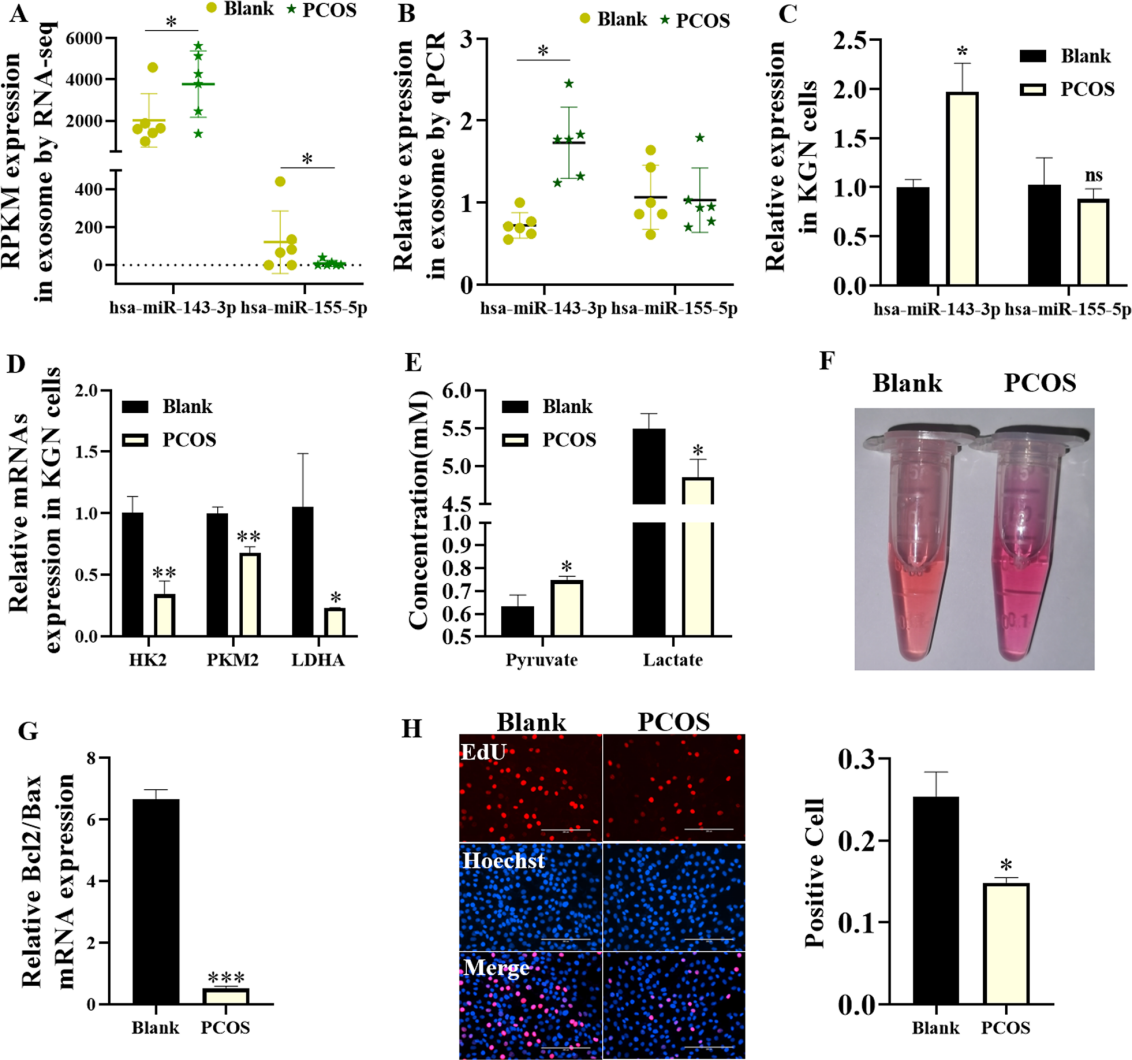
The state of KGN cells with PCOS environment changes. **A** The expression of miR-143-3p and miR-155-5p in exosomes was analysed by RNA-seq. **B** The expression of exosomal miR-143-3p and miR-155-5p was analysed by qPCR. **C** The expression of miR-143-3p and miR-155-5p in KGN cells was analysed by qPCR. **D** The expression of glycolytic DEGs (HK2, PKM2 and LDHA) in KGN cells was analysed by qPCR. **E** Concentration of glycolytic products in KGN cell culture medium. **F** Culture medium characterization of KGN cells. **G** The Bcl2/Bax expression ratio was analysed by qPCR. **H** EDU shows the cell proliferation index (*p < 0.05, **p < 0.01, ***p < 0.001)

Based on the RNA-seq analysis, we found that FF-derived exosomal miR-143-3p had upregulated expression in the PCOS group compared to the control group, while FF-derived exosomal miR-155-5p had downregulated expression in the PCOS group compared to the control group (Fig. 5A). The qRT-PCR results confirmed that FF-derived miR-143-3p but not miR-155-5p had significantly upregulated expression in the PCOS group compared to the control group (Fig. 5B). To explore the in vitro functions of miR-143-3p and miR-155-5p, we cultured KGN cells with 100 nM testosterone to mimic the in vitro cellular model of PCOS. As shown in Fig. 5C, KGN cells cultured with 100 nM testosterone (named the PCOS group) showed higher expression levels of miR-143-3p than those of cells cultured with normal full medium, while the expression level of miR-155-5p was not affected (Fig. 5C). Consistently, the expression levels of HK2, PKM2 and LDHA in the KGN cells from the PCOS group were significantly higher than those in the blank group (Fig. 5D). In addition, the concentration of pyruvate in KGN cells was significantly higher and the concentration of lactate in KGN cells was significantly lower in the PCOS group than in the blank group (Fig. 5E). The pH value of the culture medium was increased in the PCOS group compared to the blank group (Fig. 5F). The qRT-PCR results showed that the ratio of Bcl-2/Bax was significantly reduced in the KGN cells from the PCOS group compared to the blank group (Fig. 5G). In addition, the EdU assay showed that the number of EdU-positive KGN cells was significantly reduced in the PCOS group compared to the blank group (Fig. 5H).

### Effects of miR-143-3p on glycolysis in KGN cells in the PCOS groups

**Fig. 6:**
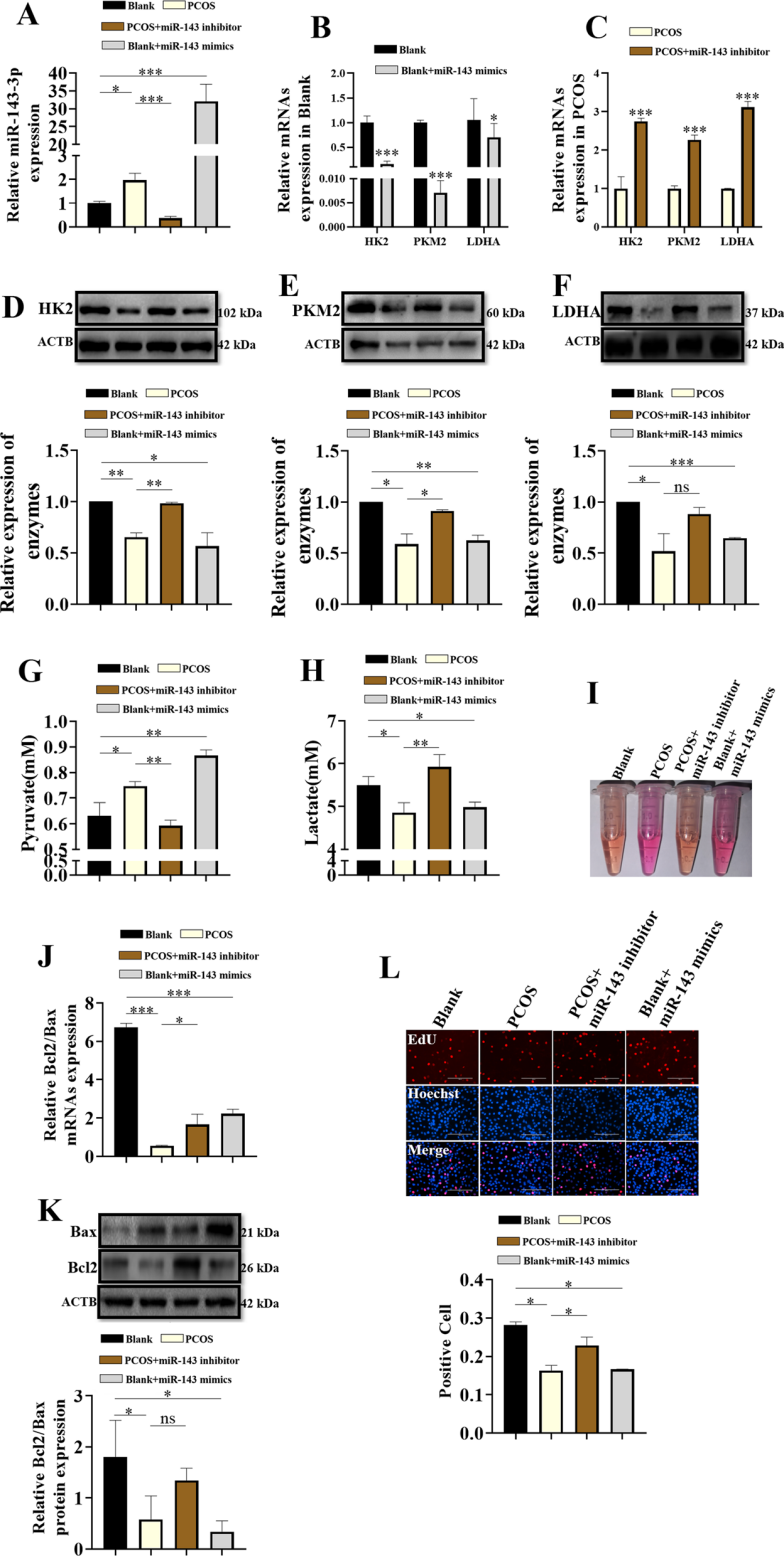
The expression changes of miR-143-3p can affect the glycolysis and proliferation of KGN cells. **A** qPCR analysis of miR-143-3p expression in cell models. **B** qPCR analysis of the expression of key DEGs (HK2, PKM2, LDHA) in cell models in a control environment. **C** qPCR analysis of the expression of key DEGs (HK2, PKM2, LDHA) in cell models in a PCOS environment. **D** Western blot analysis of HK2 in cell models. **E** Western blot analysis of PKM2 in cell models. **F** Western blot analysis of LDHA in cell models. **G** Metabolic concentration of pyruvate in cell model medium. **H** Metabolic concentration of lactate in cell model medium. **I** Culture medium characterization of the cell model. **J** qPCR analysis of Bcl2/Bax in cell models. **K** Western blot analysis of Bcl2/Bax in cell models. **L** EDU shows the proliferation index of the cell model (*p < 0.05, **p < 0.01, ***p < 0.001)

To further explore the influence of miR-143-3p on KGN cells, we performed gain-of-function and loss-of-function assays. MiR-143-3p mimic transfection significantly increased the expression level of miR-143-3p in the KGN cells cultured with full medium (Fig. 6A), while miR-143-3p inhibitor transfection significantly downregulated the expression level of miR-143-3p in the KGN cells cultured with full medium supplemented with 100 nM testosterone (Fig. 6A). Overexpression of miR-143-3p significantly downregulated the expression of HK2, LDHA and PKM2 in the blank + miR-143 mimic group compared to the blank group (Fig. 6B, D–F), while knockdown of miR-143-3p upregulated the expression of HK2, LDHA and PKM2 in the PCOS + miR-143 inhibitor group compared to the PCOS group (Fig. 6C–F). Consistently, miR-143-3p overexpression repressed the pyruvate level and increased the lactate level in the blank + miR-143 mimic group compared to the blank group (Fig. 6G, H); however, the pyruvate level was significantly higher and the lactate level was significantly lower in the PCOS + miR-143 inhibitor group than in the PCOS group (Fig. 6G, H). In addition, miR-143-3p overexpression increased the pH in the blank + miR-143 mimic group compared to the blank group (Fig. 6I), while miR-143-3p knockdown decreased the pH in the PCOS + miR-143 inhibitor group compared to the PCSO group (Fig. 6I). In addition, the qRT-PCR, Western blot and EdU assay results showed that the ratio of Bcl-2/Bax and EdU-positive cells were significantly lower in the blank + miR-143 mimic group than in the blank group (Fig. 6J–L); in contrast, the ratio of Bcl-2/Bax and EdU-positive cells were significantly higher in the PCOS + miR-143 inhibitor group than in the PCOS group (Fig. 6J–L).

### Effects of miR-155-5p on glycolysis in KGN cells in the PCOS group

**Fig. 7:**
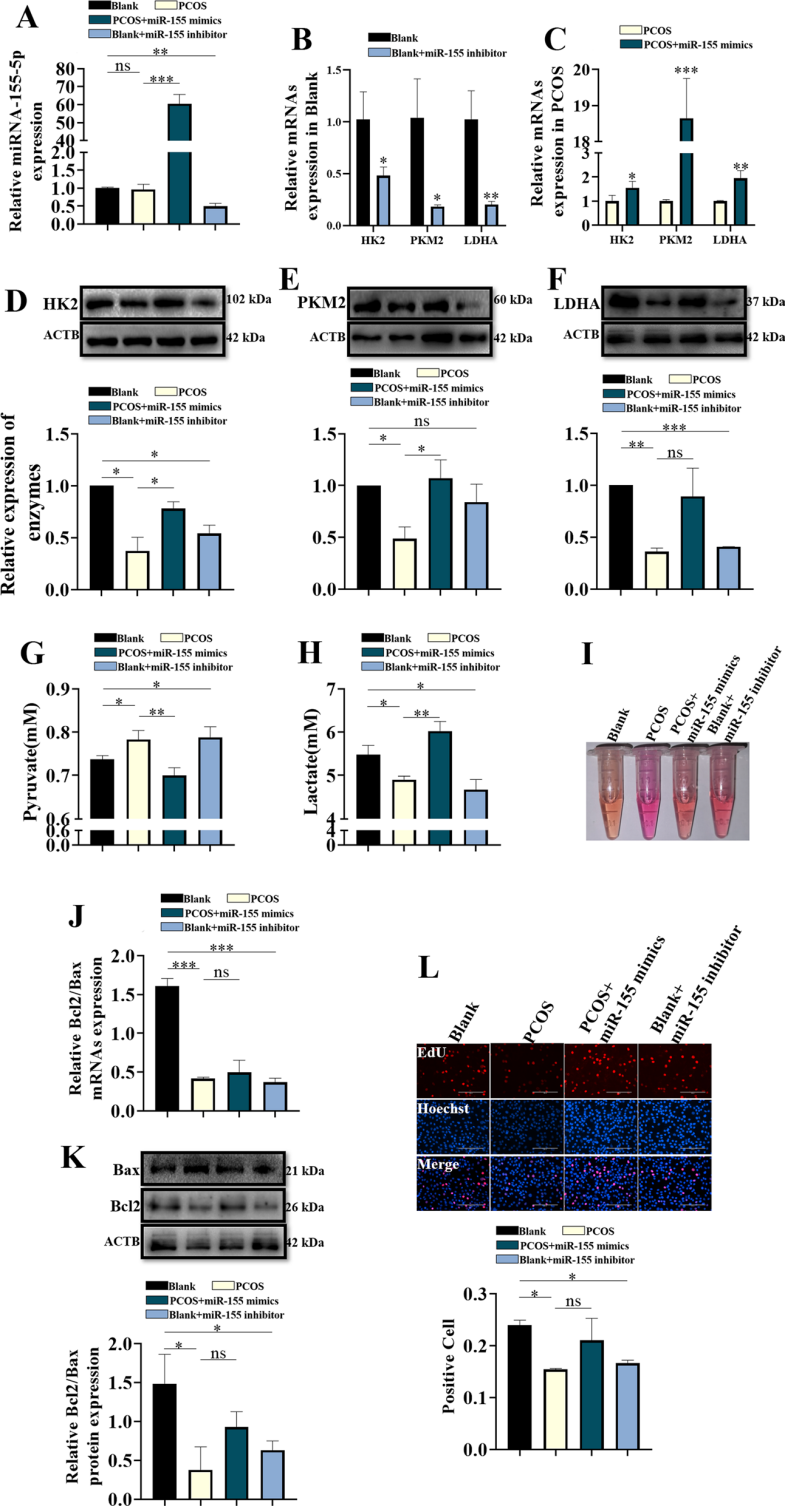
The expression changes of miR-155-5p can affect the glycolysis and proliferation of KGN cells. **A** qPCR analysis of miR-155-5p expression in cell models. **B** qPCR analysis of the expression of key DEGs (HK2, PKM2, LDHA) in cell models in a control environment. **C** qPCR analysis of the expression of key DEGs (HK2, PKM2, LDHA) in cell models in a PCOS environment. **D** Western blot analysis of HK2 in cell models. **E** Western blot analysis of PKM2 in cell models. **F** Western blot analysis of LDHA in cell models. **G** Metabolic concentration of pyruvate in cell model medium. **H** Metabolic concentration of lactate in cell model medium. **I** Culture medium characterization of the cell model. **J** qPCR analysis of Bcl2/Bax in cell models. **K** Western blot analysis of Bcl2/Bax in cell models. **L** EdU shows the proliferation index of the cell model (ns = not significant, *p < 0.05, **p < 0.01 and ***p < 0.001)

Previous studies have found that miR-155-5p may antagonize miR-143-3p in regulating glycolysis [24, 25]. We performed gain-of-function and loss-of-function assays. MiR-155-5p mimic transfection significantly increased the expression level of miR-155-5p in the KGN cells cultured with full medium with 100 nM testosterone (Fig. 7A), while miR-155-5p inhibitor transfection significantly downregulated the expression level of miR-155-5p in the KGN cells cultured with full medium (Fig. 7A). Knockdown of miR-155-5p significantly downregulated the expression of HK2, LDHA and PKM2 in the blank + miR-155 inhibitor group compared to the blank group (Fig. 7B, D–F); in contrast, miR-155-5p overexpression upregulated the expression of HK2, LDHA and PKM2 in the blank + miR-155 inhibitor group compared to the PCOS group (Fig. 7C–F).

Consistently, miR-155-5p knockdown increased the pyruvate level and decreased the lactate level in the blank + miR-155 inhibitor group compared to the blank group (Fig. 7G, H); in contrast, the pyruvate level was significantly lower and the lactate level was significantly higher in the PCOS + miR-155 mimic group than in the PCOS group (Fig. 7G, H). Furthermore, miR-155-5p knockdown increased the pH in the blank + miR-155 inhibitor group compared to the blank group (Fig. 7I), while miR-155-5p overexpression decreased the pH in the PCOS + miR-155 mimic group compared to the PCOS group (Fig. 7I). In addition, the qRT-PCR, Western blot and EdU assay results showed that the ratio of Bcl-2/Bax and EdU-positive cells were significantly lower in the blank + miR-155 inhibitor group than in the blank group (Fig. 7J–L); in contrast, the ratio of Bcl-2/Bax and EdU-positive cells were significantly higher in the PCOS + miR-155 mimic group than in the PCOS group (Fig. 7J–L).

## Discussion

**Fig. 8:**
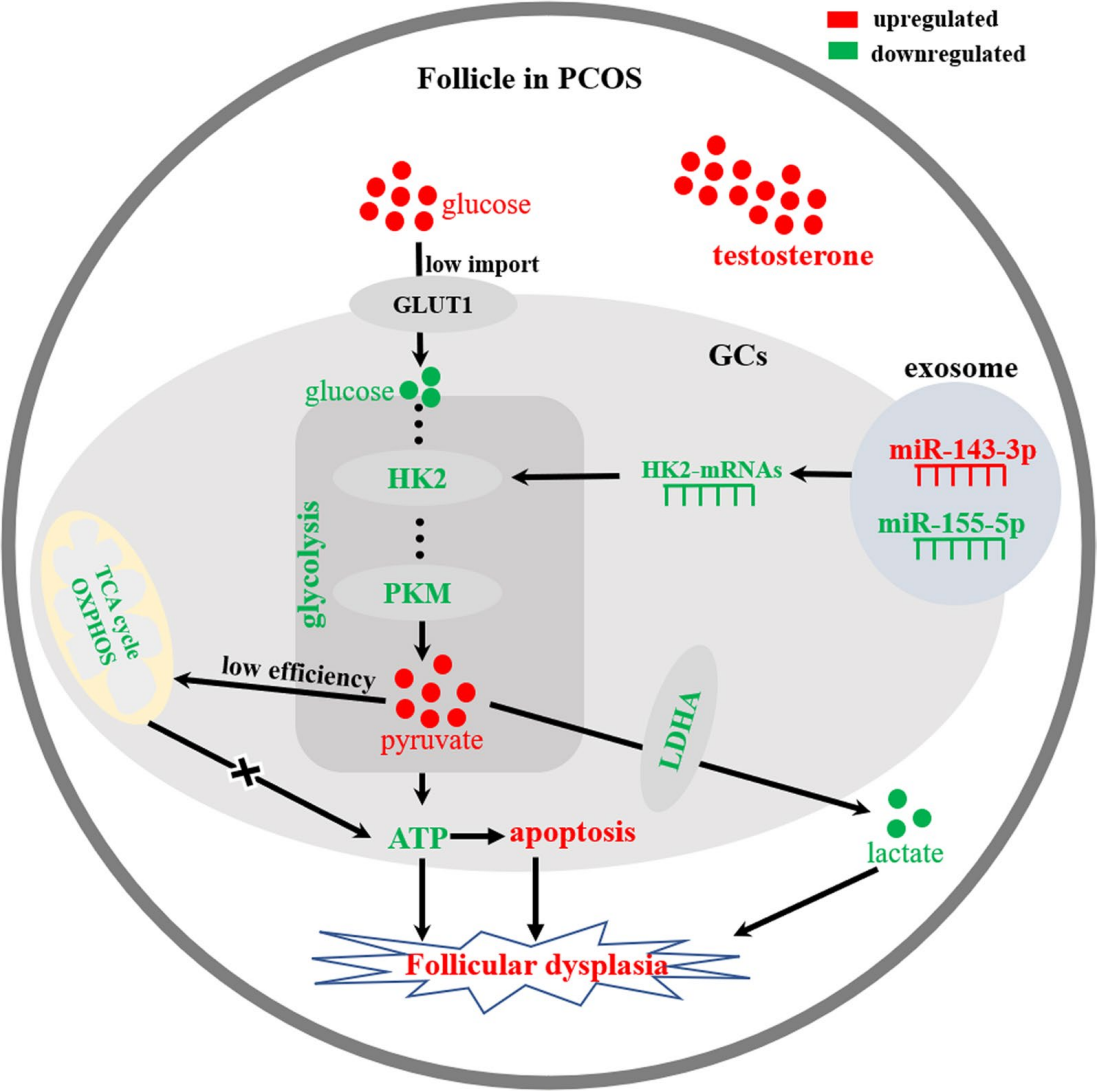
FF-derived exosomal miR-143-3p/miR-155-5p mediate follicular dysplasia by antagonizing glycolysis of GCs in PCOS

In this study, we extracted FF exosomes from patients in the control group (n = 6) and PCOS group (n = 6) and identified their quality. By RNA-seq of exosomes, we found that miR-143-3p and miR-155-5p could potentially regulate the glycolysis of GCs through HK2. KGN cells were used to verify this potential regulation in vitro. We found that compared with those of the control group, the follicular GCs of the patients with PCOS were in a high-testosterone and high-glucose environment, and the upregulation of miR-143-3p expression and downregulation of miR-155-5p expression in exosomes antagonized the glycolytic process of GCs, resulting in an accumulation of pyruvate, a decrease in lactate production, and a decrease in the energy metabolism of GCs. The above processes lead to apoptosis and follicular dysplasia (Fig. 8). Exosomes are extracellular vesicles secreted by exocytosis [26] and can be identified by the expression of the CD9, CD63, and CD81 proteins. Exosomes are cup-shaped with a diameter of 30–200 nm under TEM [27, 28], which is consistent with our results for the FF-derived exosomes.

Exosomes contain a large number of miRNAs and other substances, and miRNAs perform functions through the intercellular transfer of exosomes [29, 30]. With the development and expansion of exosomal miRNA-based cancer treatment and exosomal inhibitors [13, 31, 32], elucidation of regulation of PCOS based on FF-derived exosomal miRNAs is urgently needed. In RNA-seq, we identified biological processes such as glucose import and utilization, glycolysis, protein kinase synthesis, ATP synthesis and apoptosis of GCs regulated by exosome miRNA. Increasing evidence has shown that miRNAs control the expression of key glycolytic enzymes, including HK2, PKM2 and LDHA, to regulate glycolysis through the Warburg effect in cells [33–35]. In PCOS, exosomal miR-143-3p and miR-155-5p antagonize glycolysis in KGN cells by silencing key enzymes. The overall level of glycolysis is decreased. ATP generation during the mid-glycolysis period is reduced, resulting in insufficient energy supply for follicular development, which is the primary factor mediating PCOS-related follicular dysplasia in this study.

The level of lactate, a metabolite of glycolysis, has always been used as a marker to measure the metabolic level of the Warburg effect in cells [36]. Studies have found that the follicular development of patients with PCOS requires a high concentration of lactate stimulation [37]. Pyruvate accumulation and a decrease in lactate in the culture medium in the PCOS group were confirmed in the metabolome of the rat model of PCOS [38]. The above shows that in PCOS, exosomal miR-143-3p and miR-155-5p silence glycolysis in KGN cells, which leads to a decrease in lactate levels, thereby weakening the environmental stimulation of follicular development. This phenomenon is the second factor that mediates PCOS follicular dysplasia in this study. In addition, apoptosis of GCs is considered the main mechanism of follicular atresia [39]. Upregulation of PCOS FF-derived exosomal miR-143-3p expression and downregulation of miR-155-5p expression caused apoptosis of GCs by antagonizing glycolysis, which finally mediated PCOS-related follicular dysplasia in this study.

MiR-143-3p has been confirmed to negatively regulate glycolysis by targeting HK2 in a variety of cells [40–42]. HK2-associated cellular glycolysis is thought to be involved in the coregulation of miR-143-3p and miR-155-5p [25]. In PCOS, HK2 mediates glycolysis in KGN cells mediated by miR-143-3p and miR-155-5p, which is similar to the role of HK2 in breast cancer cells [24]. This finding suggests that HK2 may be one of the keys to PCOS therapy and suggests a direction for us to explore PCOS in the future. MiR-143-3p plays a role in the clinic mainly based on its molecular mechanism of targeting HK2 to regulate glycolytic energy metabolism in cells, which has been widely confirmed in colon cancer cells, oral squamous cell carcinoma cells, and other cells [40, 41]. This regulatory mechanism is the same as that found in KGN cells. Our results suggest that miR-143-3p can provide a theoretical basis and technical method for the GC-based glycolytic pathway of PCOS treatment and efficacy evaluation of its specific visual dynamic.

The positive regulation of glycolysis by miR-155-5p has been continuously supported by clinical and epidemiological studies [43, 44]. In this study, downregulated miR-155-5p expression weakened glycolysis in KGN cells and indirectly promoted cell apoptosis. Overexpression of miR-155-5p in the PCOS environment could significantly activate glycolysis and proliferation in KGN cells, and miR-155-5p positively regulated glycolysis in KGN cells, providing a new strategy for alleviation of PCOS. We found that miR-155-5p expression was significantly downregulated in RNA-seq of PCOS exosomes but not significantly downregulated in qPCR of KGN cells and exosomes. We speculated that the cascade reaction between miR-155-5p and miR-143-3p may amplify the regulation of miR-155-5p on glycolysis in KGN cells, which is caused by the low accuracy of qPCR. The amplification effect of the cascade reaction suggests that a slight increase in the expression of miR-155-5p in exosomes from individuals with PCOS may activate glycolysis in GCs.

In clinical samples of exosomes, the expression of miR-143-3p in the PCOS group was up to ten times higher than that in the control group, showing strong inhibition of glycolysis in the patients with PCOS. MiR-155-5p was expressed in the control group but not in some samples of the PCOS group. Compared with the strong inhibition of miR-143-3p on glycolysis, miR-155-5p has little positive regulation on glycolysis in patients with PCOS. Therefore, we proposed that in the antagonistic relationship between miR-143-3p and miR-155-5p, miR-143-3p predominantly mediated the decrease in glycolysis in follicular GCs of patients with PCOS, and miR-155-5p predominantly maintained normal glycolysis in follicular GCs in subjects without PCOS. MiR-143-3p and miR-155-5p play different roles in different environments and have opposite regulatory effects on glycolysis in follicular GCs, which reveals the specific mechanism by which exosomal miR-143-3p and miR-155-5p antagonize glycolysis mediating follicular dysplasia of GCs. This finding also suggests that clinical detection of miR-143-3p and miR-155-5p levels may be used for early prediction of PCOS.

In this study, we reveal the proposed potential therapeutic targets and mechanisms by which exosomal miR-143-3p and miR-155-5p antagonize glycolysis in PCOS-related follicular dysplasia. In this study, the findings of the FF-derived exosomal transcriptome were confirmed through cell experiments and in-depth exploration. The consistency of the results in clinical samples and experimental detection further highlights the clinical applicability and reliability of this study.

## Data Availability

The datasets generated and/or analyzed during the current study are available from the corresponding author on reasonable request.
