# Correction: Follicular fluid-derived exosomal miR-143-3p/miR-155-5p regulate follicular dysplasia by modulating glycolysis in granulosa cells in polycystic ovary syndrome

**DOI:** 10.1186/s12964-022-00938-9

**Published:** 2022-08-01

**Authors:** Jianping Cao, Peng Huo, Kuiqing Cui, Huimei Wei, Junna Cao, Jinyuan Wang, Qingyou Liu, Xiaocan Lei, Shun Zhang

**Affiliations:** 1 Guangxi Key Laboratory of Environmental Exposomics and Entire Lifecycle Health, Guilin Medical University, Guilin, 541199 China; 2 State Key Laboratory for Conversation and Utilization of Subtropical Agro-Bioresources, Guangxi University, Nanning, 530004 Guangxi China; 3 Department of Reproductive Medical Center, The Affiliated Hospital of Guilin Medical University, Guilin, 541001 China; 4 Department of Histology and Embryology, Clinical Anatomy and Reproductive Medicine Application Institute, University of South China, Hengyang, 421001 China

## Correction to: Cell Communication and Signaling(2022)20:61 10.1186/s12964-022-00876-6

Following publication of the original article [1], the authors identified an omission in the Funding section. The funding section should read as follows:

The present study was supported by China National Natural Science Fund (Nos. 81960274, 82160289, 82101720 and 32160790), Guangxi Bagui Scholar Program, and Guangxi Science Foundation (Nos. 2020GXNSFAA297097 and 2018GXNSFDA050003).

The original article [1] has been updated.
